# Supplementary material for: A Critical View of the Application of the APEX Software (Aqueous Photochemistry of Environmentally-Occurring Xenobiotics) to Predict Photoreaction Kinetics in Surface Freshwaters
Source: Molecules. 2019 Dec 18;25(1):9. doi: 10.3390/molecules25010009 (PMC7017383; doi:10.3390/molecules25010009)
Supplement: Supplementary file 1 [file molecules-25-00009-s001.zip › 3.2.4_gcc-4.4.0.zip!/3.2.4_gcc-4.4.0/3.2.4_gcc-4.4.0/doc/octave/html/interpreter/Identifying-points-in-Triangulation.html]

Identifying points in Triangulation - Untitled


Previous: Plotting the Triangulation,
Up: Delaunay Triangulation


---

#### 29.1.2 Identifying points in Triangulation

It is often necessary to identify whether a particular point in the
N-dimensional space is within the Delaunay tessellation of a set of
points in this N-dimensional space, and if so which N-simplex contains
the point and which point in the tessellation is closest to the desired
point. The functions `tsearch` and `dsearch` perform this
function in a triangulation, and `tsearchn` and `dsearchn` in
an N-dimensional tessellation.

To identify whether a particular point represented by a vector p
falls within one of the simplices of an N-simplex, we can write the
Cartesian coordinates of the point in a parametric form with respect to
the N-simplex. This parametric form is called the Barycentric
Coordinates of the point. If the points defining the N-simplex are given
by N `+ 1` vectors t(i,:), then the Barycentric
coordinates defining the point p are given by

```
     p = sum (beta(1:N+1) * t(1:N+1),:)
```

where there are N `+ 1` values beta`(`i`)`
that together as a vector represent the Barycentric coordinates of the
point p. To ensure a unique solution for the values of
beta`(`i`)` an additional criteria of

```
     sum (beta(1:N+1)) == 1
```

is imposed, and we can therefore write the above as

```
     p - t(end, :) = beta(1:end-1) * (t(1:end-1, :)
           - ones(N, 1) * t(end, :)
```

Solving for beta we can then write

```
     beta(1:end-1) = (p - t(end, :)) / (t(1:end-1, :)
           - ones(N, 1) * t(end, :))
     beta(end) = sum(beta(1:end-1))
```

which gives the formula for the conversion of the Cartesian coordinates
of the point p to the Barycentric coordinates beta. An
important property of the Barycentric coordinates is that for all points
in the N-simplex

```
     0 <= beta(i) <= 1
```

Therefore, the test in `tsearch` and `tsearchn` essentially
only needs to express each point in terms of the Barycentric coordinates
of each of the simplices of the N-simplex and test the values of
beta. This is exactly the implementation used in
`tsearchn`. `tsearch` is optimized for 2-dimensions and the
Barycentric coordinates are not explicitly formed.

— Loadable Function: idx = **tsearch** (x, y, t, xi, yi)  
> Searches for the enclosing Delaunay convex hull. For t `=
> delaunay (`x`,` y`)`, finds the index in t containing the
> points `(`xi`,` yi`)`. For points outside the convex hull,
> idx is NaN.
>
>
>
> **See also:** delaunay, delaunayn.

— Function File: [idx, p] = **tsearchn** (x, t, xi)  
> Searches for the enclosing Delaunay convex hull. For t `=
> delaunayn (`x`)`, finds the index in t containing the
> points xi. For points outside the convex hull, idx is NaN.
> If requested `tsearchn` also returns the Barycentric coordinates p
> of the enclosing triangles.
>
>
>
> **See also:** delaunay, delaunayn.

An example of the use of `tsearch` can be seen with the simple
triangulation

```
     x = [-1; -1; 1; 1];
     y = [-1; 1; -1; 1];
     tri = [1, 2, 3; 2, 3, 1];
```

consisting of two triangles defined by tri. We can then identify
which triangle a point falls in like

```
     tsearch (x, y, tri, -0.5, -0.5)
     => 1
     tsearch (x, y, tri, 0.5, 0.5)
     => 2
```

and we can confirm that a point doesn't lie within one of the triangles like

```
     tsearch (x, y, tri, 2, 2)
     => NaN
```

The `dsearch` and `dsearchn` find the closest point in a
tessellation to the desired point. The desired point does not
necessarily have to be in the tessellation, and even if it the returned
point of the tessellation does not have to be one of the vertexes of the
N-simplex within which the desired point is found.

— Function File: idx = **dsearch** (x, y, tri, xi, yi)  
— Function File: idx = **dsearch** (x, y, tri, xi, yi, s)  
> Returns the index idx or the closest point in x`,` y
> to the elements `[`xi`(:),` yi`(:)]`. The variable s is
> accepted but ignored for compatibility.
>
>
>
> **See also:** dsearchn, tsearch.

— Function File: idx = **dsearchn** (x, tri, xi)  
— Function File: idx = **dsearchn** (x, tri, xi, outval)  
— Function File: idx = **dsearchn** (x, xi)  
— Function File: [idx, d] = **dsearchn** (...)  
> Returns the index idx or the closest point in x to the elements
> xi. If outval is supplied, then the values of xi that are
> not contained within one of the simplicies tri are set to
> outval. Generally, tri is returned from `delaunayn
> (`x`)`.
>
>
>
> **See also:** dsearch, tsearch.

An example of the use of `dsearch`, using the above values of
x, y and tri is

```
     dsearch (x, y, tri, -2, -2)
     => 1
```

If you wish the points that are outside the tessellation to be flagged,
then `dsearchn` can be used as

```
     dsearchn ([x, y], tri, [-2, -2], NaN)
     => NaN
     dsearchn ([x, y], tri, [-0.5, -0.5], NaN)
     => 1
```

where the point outside the tessellation are then flagged with `NaN`.
